# Argon gas poisoning leading to persistent memory impairment: A 2-year case report

**DOI:** 10.1097/MD.0000000000038545

**Published:** 2024-06-14

**Authors:** Weiwei Gao, Jingjing She, Mingyang Wang, Shuixian Li, Xingyu Chen, Renjing Zhu

**Affiliations:** a Department of Neurology, Zhongshan Hospital of Xiamen University, School of Medicine, Xiamen University, Xiamen, China; b Department of Neurology, Jimusaer County People’s Hospital, China.

**Keywords:** argon gas, asphyxiant gas, case report, hippocampal deficit, poisoning, β-amyloid peptide

## Abstract

**Rationale:**

Argon gas poisoning is an often overlooked yet critical public health concern with the potential for severe and persistent neurological consequences. Current treatment protocols primarily focus on acute-phase management, but a comprehensive understanding of the long-term neurological effects remains incomplete.

**Patient concerns:**

A 22-year-old male worker was found unconscious in the furnace room of an argon production facility. After regaining consciousness, he presented with symptoms of dizziness, headache, fatigue, and irritability. Neurological examination revealed impairments in both recent and remote memory, notably pronounced short-term memory deficits and reduced arithmetic skills.

**Diagnosis:**

Argon gas poisoning, hypoxic encephalopathy, and mild hepatic and renal dysfunction.

**Interventions:**

Upon admission, symptomatic supportive measures included oxygen therapy via nasal cannula (3 L/min), daily hyperbaric oxygen therapy (1.5 ATA, 60 minutes), oral neurotrophic methylcobalamin (0.5 mg, 3 times daily), and intravenous vitamin C infusion (2 g daily) to scavenge oxygen free radicals.

**Outcome:**

A 2-year telephone follow-up indicated persistent short-term memory impairment, particularly with memorizing numbers. In a memory test, he achieved a digit span forward of 5 but a digit span backward of 2, indicating impairment. Despite these challenges, his daily life and work performance remained largely unaffected.

**Lesson:**

This case offers valuable insights into the biological mechanisms underlying prolonged neurological sequelae following asphyxiating gas exposure, specifically the persistent impairment of hippocampal function.

## 1. Introduction

Asphyxiating gases such as nitrogen, argon, and helium are relatively nontoxic but can cause tissue hypoxia and subsequent asphyxiation. Inhaling high concentrations or large volumes of these gases disrupts the body oxygen supply, uptake, transport, and utilization, leading to reduced arterial hemoglobin oxygen saturation and partial pressure, ultimately resulting in asphyxiating gas poisoning. These gases are inert and odorless, making detection challenging, particularly in enclosed spaces where dangerous levels can accumulate. Additionally, inhalation of these inert gases promotes excessive exhalation of carbon dioxide, which reduces stimulation of the respiratory center and diminishes early warning signs of hypoxia, increasing the abruptness and risk of poisoning.^[1]^

Argon, comprising 0.93% of atmospheric air, is the most abundant inert gas. Although inherently colorless, odorless, and nontoxic, argon at concentrations exceeding 33% significantly reduces the partial pressure of oxygen in the air, leading to hypoxic asphyxiation. We report a case of acute argon poisoning at an argon production facility where the patient developed persistent hippocampal deficits characterized by anterograde amnesia lasting for years.

## 2. Case presentation

A 22-year-old male worker was found unconscious in the furnace room of an argon production plant at 4:50 am on August 19, 2021. He had last been seen in a normal state at 4:20 am He was immediately removed from the furnace room, given positive pressure ventilation, and was urgently transported to a nearby county hospital. After approximately 30 minutes, he regained partial consciousness and attempted to communicate, but his responses were inaccurate. As he became more alert, he complained of dizziness, headache, fatigue, and irritability.

One hour later, the patient was transferred to our hospital emergency department. Initial physical examination revealed stable vital signs with no abnormalities in the pulmonary, abdominal, or cardiac system. Neurological examination revealed that he was alert but somewhat slow to respond and not very talkative. While comprehension and expression were normal, he exhibited impaired recent and remote memory, significant short-term memory loss, and reduced sequential calculation ability. His orientation, cranial nerves, motor and sensory systems, and pathological reflexes were normal. He had no significant personal or family medical history.

## 3. Investigations

**Figure 1.**
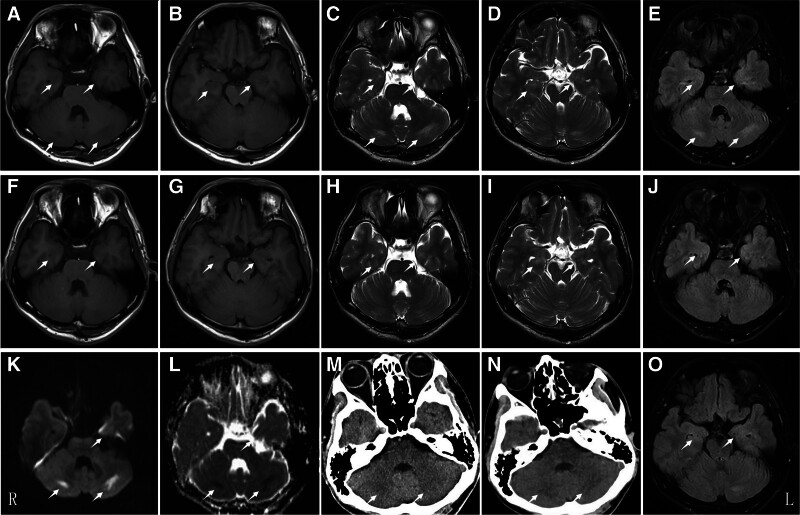
Cranial MRI (A–J,O) 29 hours after onset revealed bilateral cerebellum and hippocampus with low signal in T1 and ADC and high signal in T2, T2-FLAIR and DWI (white arrows). Follow-up brain MRI (F–E,K,L) showed slightly low signal in T1, high signal in T2, and T2-FLAIR in bilateral hippocampus 4 mo later. Cranial CT 19 h after onset (N) showed more visible and lower signal than that 1.5 h after onset (M). ADC = apparent diffusion coefficient, DWI = diffusion-weighted inversion recovery, MRI = magnetic resonance imaging.

Laboratory investigations conducted 70 minutes after symptom onset revealed elevated markers for liver and renal function, as well as muscle tissue injury. Blood glucose was also elevated, indicating mild multiorgan injury (Table 1). These markers decreased after 1 hour of follow-up. Routine blood tests revealed elevated leukocyte counts that continued to rise in the following hour, indicating an inflammatory response. Venous blood potassium and sulfur levels, arterial blood gas analysis, coagulation profile, and a 12-lead electrocardiogram were all within normal limits. A cranial computed tomography (CT) scan taken 19 hours after onset revealed prominent changes in the cerebellar region compared to the initial scan taken 1.5 hours postonset. At 29 hours postonset, cranial magnetic resonance imaging (MRI) revealed low T1 and apparent diffusion coefficient (ADC) signals and high T2, T2-FLAIR, and diffusion-weighted inversion recovery (DWI) signals in the bilateral cerebellar and hippocampal regions (Fig. 1).

**Table 1:** The patient multiple laboratory indicators.

Laboratory test	WBC (10^9^/L)	AST (U/L)	ALT (U/L)	ALP (U/L)	GGT (U/L)	CREA (µmol/L)	BG (mmol/L)	LDH (U/L)	CK (U/L)	CK-MB (U/L)
First (70 min)	**10.1**	**304**	**232**	**126**	**68**	**120**	**12.65**	**545.3**	**262.9**	**35.03**
Second (3 h)	**10.9**	**262**	**213**	104	**61**	85	**9.9**	**497.4**	**364.3**	23.32
Third (19 h)	6.86	ND	ND	ND	ND	ND	ND	**323.0**	**1107**	**40**
Reference range	4–10	8–40	0–40	15–112	53–97	53–97	3.9–6.2	114–20	38–174	0–25

Bold: above normal.

ALP = alkaline phosphatase, ALT = alanine aminotransferase, AST = aspartate aminotransferase, BG = blood glucose, CK = creatine kinase, CKMB = creatine kinase myocardial band, CREA = creatinine, GGT = gamma-glutamyltransferase, LDH = lactate dehydrogenase, LT = Laboratory test, ND = not done, WBC = white blood cell. The first 2 times of blood sampling was carried out on an empty stomach.

## 4. Differential diagnosis

Before attributing the loss of consciousness to argon poisoning, a comprehensive assessment of other potential causes, including carbon monoxide poisoning, chemical exposure, cerebrovascular events, diabetic ketoacidosis, and seizures, should be conducted.

Carbon Monoxide Poisoning: Investigate exposure to sources of incomplete fuel combustion, such as gas or charcoal-based heating and cooking equipment. Confirm the diagnosis by measuring blood carboxyhemoglobin levels.

Other Chemical Exposures: A detailed history of occupational, environmental, and chemical exposure was obtained. No specific chemical exposure history has been reported.

Cerebrovascular Events: The patient did not exhibit hallmark stroke symptoms such as hemiparesis, speech disorders, or facial muscle weakness. Normal CT and MRI head scans ruled out cerebrovascular events.

Diabetes Ketoacidosis: The patient had no known history of diabetes mellitus and did not exhibit symptoms such as excessive thirst, polyuria, or fruity breath. Blood glucose, ketone bodies, and blood gas analyses were all within normal ranges, excluding diabetic ketoacidosis.

## 5. Treatment

Upon admission, the patient received oxygen therapy via nasal cannula (3 L/min) and daily hyperbaric oxygen therapy (1.5 ATA for 60 minutes). Supportive therapy included oral methylcobalamin (0.5 mg, 3 times daily) and intravenous vitamin C (2 g daily). Two days later, he was transferred to a higher-level hospital for continued nasal cannula oxygen (3 L/min), daily hyperbaric oxygen therapy, oral methylcobalamin (0.5 mg, 3 times daily), and ginkgo biloba extract (40 mg, 3 times daily). After 10 days of comprehensive treatment, his level of consciousness stabilized, and his memory impairment improved significantly. He was discharged without further medication.

## 6. Outcome and follow-up

At a follow-up MRI 4 months after discharge, all abnormal cerebellar signals had resolved (Fig. 1). However, T1 signals remained decreased, and T2 signals increased in the bilateral hippocampus. One year later, a follow-up telephone interview revealed that the patient still experienced mild memory impairment but no longer had significant disruptions in daily life. During the 2-year follow-up (January 5, 2024), he reported persistent difficulty recalling numbers. In a memory test, he achieved a digit span forward of 5 but a digit span backward of 2, indicating impairment. Despite these challenges, his daily life and work performance remained largely unaffected.

## 7. Discussion

In recent years, there has been growing interest in argon due to its distinctive neuroprotective properties.^[2]^ Various in vivo and ex vivo studies, including oxygen-glucose deprivation cell culture experiments, transient middle cerebral artery occlusion models, and retinal ischemia-reperfusion injury models, have demonstrated the significant impact of argon on improving neuronal function and cell survival.^[3]^ However, Anke et al reported that the effects of argon on the nervous system might be dualistic, depending on concentration and exposure duration.^[4]^ When the argon concentration in the air exceeds 33%, the oxygen level significantly decreases, reducing gas diffusion across the alveolar-capillary membrane and impairing the gas exchange process. This ultimately leads to inadequate oxygen absorption from the inhaled air and hypoxic asphyxia.

Argon-induced asphyxiation primarily occurs due to cellular hypoxia. Acute hypoxia disrupts the mitochondrial electron transport chain, leading to increased electron leakage and overproduction of reactive oxygen species (ROS), such as superoxide anion radicals. Hypoxia also activates ROS-generating pathways, including NADPH oxidase and xanthine oxidase pathways. These reactive and toxic molecules attack essential cellular components such as lipids, proteins, and DNA, compromising cellular structural integrity and function. The resulting oxidative stress ultimately causes cellular damage, leading to apoptosis and necrosis.^[5]^

The brain has a uniquely high metabolic rate and oxygen consumption but low antioxidant enzyme activity, making it much more dependent on oxygen than other tissues and organs.^[6]^ In this patient, blood tests taken at 70 minutes and 3 hours postonset indicated mild liver, kidney, and muscle damage. Notably, a CT scan performed 19 hours after onset showed more pronounced low signals than did an earlier scan taken 1.5 hours after onset. An MRI scan at 29 hours postonset demonstrated low T1 and ADC signals, as well as high T2, T2-FLAIR, and DWI signals in the bilateral cerebellar and hippocampal regions. An MRI follow-up conducted 4 months later revealed slightly decreased T1 and increased T2 signals in the bilateral hippocampus (Fig. 1). These imaging findings suggest that despite the patient rapid recovery after treatment, acute hypoxia caused more severe and lasting damage in the hippocampus than in the cerebellum, even though the initial imaging changes were mild.

The cornu ammonis 1 (CA) region of the hippocampus plays a crucial role in memory formation and consolidation.^[7]^ Previous studies have shown that delayed neuronal death (DND) in the CA1 region after acute hypoxia is strongly associated with memory deficits.^[8]^ We hypothesize that this patient hippocampus, specifically the CA1 region, triggered DND following acute hypoxia, leading to persistent anterograde amnesia symptoms observed even 2 years later.

The susceptibility of brain regions to hypoxia varies across regions, with the hippocampus, particularly the CA1 region, being especially vulnerable.^[1,9]^ This sensitivity is influenced by hypoxemia, β-amyloid peptide (β-AP) neurotoxicity, and ischemia-induced metabolic and oxidative stress.^[10]^ After acute hypoxic injury, different hippocampal regions exhibit distinct pathological changes: the CA4 region shows acute ischemic cellular alterations, while the CA1 region undergoes gradual, widespread neuronal loss.^[11]^ Beilharz et al demonstrated that hippocampal neuronal damage after ischemia-hypoxia occurs through both necrotic and apoptotic pathways. In the acute phase, hypoxia disrupts metabolism, intracellular ion balance, and the oxidative system, leading to acute necrosis. In contrast, neurons in regions of delayed injury gradually undergo apoptosis, particularly affecting CA1 neurons. These findings highlight that hippocampal neuronal damage extends beyond the acute phase.^[12,13]^

To investigate the potential mechanisms underlying DND, numerous studies worldwide have proposed hypotheses such as excitatory amino acid toxicity, abnormal heat shock protein expression, and impaired mitochondrial DNA expression.^[14,15]^ However, none have fully elucidated this phenomenon. Recent research on the relationship between serum β-AP and DND has garnered significant interest. Claudio et al found that patients with Alzheimer disease exhibit blood–brain barrier (BBB) disruption in regions of neuronal loss, allowing serum β-AP to cross the damaged barrier and accumulate in the brain parenchyma.^[16]^ Similar microstructural changes have been documented in animal models of acute ischemia and hypoxia.^[17]^ These leaked serum β-AP, predominantly deposited in the CA1, CA4, and dentate gyrus regions of the hippocampus, may initiate DND through multiple pathways, including oxidative stress, mitochondrial dysfunction, inflammatory responses, tau protein hyperphosphorylation, synaptic plasticity impairment, signaling disruption, and endoplasmic reticulum stress. These interconnected mechanisms can create a vicious cycle that ultimately leads to irreversible neuronal loss.^[17–23]^

β-AP disrupts the mitochondrial electron transport chain, reducing ATP synthesis and increasing ROS production through NADPH oxidase activation. This leads to lipid peroxidation, protein oxidation, and DNA damage, culminating in neuronal death.^[19]^ β-AP also causes tau protein hyperphosphorylation by activating kinases, leading to microtubule detachment and neurofibrillary tangle formation, which compromises the neuronal cytoskeleton.^[20]^ By binding to presynaptic receptors such as α7 nicotinic acetylcholine receptors and mGluR5, β-AP interferes with neurotransmitter release and synaptic transmission, resulting in synapse loss and long-term neuronal atrophy. Additionally, β-AP accumulates in the endoplasmic reticulum, causing stress and triggering the unfolded protein response.^[21]^ This process upregulates proapoptotic factors such as C/EBP homologous proteins, initiating apoptosis.^[22]^ β-AP also activates microglia and astrocytes, releasing proinflammatory cytokines (IL-1β and TNF-α) and inflammatory mediators, which directly damage neurons and exacerbate oxidative stress and mitochondrial dysfunction.^[23]^

A rat model of hippocampal ischemia showed that BBB damage can persist for 6 months after acute ischemic injury. Once the BBB is compromised, cytotoxic amyloid precursor proteins leak into the CA1 region and other hippocampal regions, exacerbating DND.^[17]^ We hypothesize that acute hypoxia caused by excessive argon inhalation causes prolonged BBB damage in the hippocampus, leading to serum β-AP deposition and DND in the CA1 region. BBB disruption is recognized as a critical early feature of hippocampal white matter lesions. This process is often followed by extensive demyelination and retrograde neuronal death, ultimately resulting in neuronal loss in the CA1 region and other hippocampal regions.

In summary, while early imaging reveals only minor changes, the hippocampus, particularly the CA1 region, sustains more severe and persistent damage than other brain structures. Although the link between DND and acute ischemia in the CA1 region of the rodent hippocampus is well documented, further research is needed to elucidate the precise neurobiological mechanisms by which acute hypoxic asphyxia alone induces DND in the hippocampus.

## 8. Learning points/take home messages

Argon-induced asphyxiating gas poisoning may cause prolonged damage to the blood-brain barrier in the hippocampus, resulting in serum β-amyloid protein deposition. This deposition can trigger DND in the CA1 region through various mechanisms.

During acute management of asphyxiating gas poisoning, clinicians must remain vigilant about its potential long-term health effects.

## Acknowledgments

The authors thank the patient for participating in this study and the medical staff involved in the patient diagnosis and treatment.

## Author contributions

**Conceptualization:** Jingjing She, Renjing Zhu

**Investigation:** Weiwei Gao, Mingyang Wang

**Methodology:** Shuixian Li, Renjing Zhu

**Supervision:** Shuixian Li, Xingyu Chen

**Writing – original draft:** Weiwei Gao, Jingjing She

**Writing – review & editing:** Mingyang Wang, Shuixian Li, Xingyu Chen, Renjing Zhu
